# Virulence Factors of *Sporothrix schenckii*

**DOI:** 10.3390/jof8030318

**Published:** 2022-03-19

**Authors:** Laura Cristina García-Carnero, José Ascención Martínez-Álvarez

**Affiliations:** Departmet of Biology, Exact and Natural Sciences Division, Universidad de Guanajuato, Noria Alta s/n, Col. Noria Alta, Guanajuato 36050, Mexico; laura.garcia@ugto.mx

**Keywords:** *Sporothrix schenckii*, virulence factors, extracellular vesicles, pathogenesis

## Abstract

*Sporothrix schenckii* is one of the etiological agents of sporotrichosis. In this review, we discuss the virulence factors that have been proven to participate in the *S. schenckii*-host interaction. Among these known factors, we can find cell wall glycoproteins, adhesins, melanin, extracellular vesicles, and dimorphism. Furthermore, the morphological transition of *S. schenckii* in response to environmental conditions such as pH and temperature represents a means by which the fungus is able to establish mycosis in mammals. One of the key features in the development of sporotrichosis is the adhesion of the fungus to the host extracellular matrix. This event represents the first step to developing the mycosis, which involves adhesins such as the glycoproteins Gp70, Hsp60, and Pap1, which play a key role during the infection. The production of melanin helps the fungus to survive longer in the tissues and to neutralize or diminish many of the host’s attacks, which is why it is also considered a key factor in pathogenesis. Today, the study of human fungal pathogens’ virulence factors is a thriving area of research. Although we know some of the virulence factors in *S. schenckii*, much remains to be understood about the complex process of sporotrichosis development and the factors involved during the infection.

## 1. Introduction

Sporotrichosis is a worldwide distributed mycosis, mainly found in tropical and subtropical areas, caused by the species of the *Sporothrix* pathogenic clade, including *Sporothrix schenckii*, *Sporothrix brasiliensis,* and *Sporothrix globosa* [1,2]; *S. schenckii* and *S. brasiliensis* are considered to be sister species, and their clade is considered to be closely related to that of *S. globosa*, which suggests that they share a more recent common ancestor than the species of the environmental clade [3]. These organisms are thermodimorphic fungi found in soil, plant organic matter, and decomposing organic matter in nature, as the mycelial or saprophytic phase, while they are found in the host as yeasts, also known as the parasitic phase [4]. The infection transmission is through traumatic inoculation into the skin with contaminated material or through zoonosis, with cats as the main vector, and while the lesions are usually restricted to the skin, several clinical forms can be developed, which range from the most commonly found lymphocutaneous and fixed cutaneous forms, to the extracutaneous and disseminated forms, present mainly in immunocompromised patients [5]. In addition, the infection can also be acquired through spore inhalation, affecting mainly the lungs [6].

Although the first report of sporotrichosis dates back to 1898 by Benjamin R. Schenck [7], and even though the genome sequencing of the three main *Sporothrix* pathogenic species is already available [8,9] and the transcriptomic data from both *S. schenckii* morphologies have been reported (http://sporothrixgenomedatabase.unime.it/, accessed on 13 February 2022), limited information about this fungus virulence factors is known [10].

**Figure 1:**
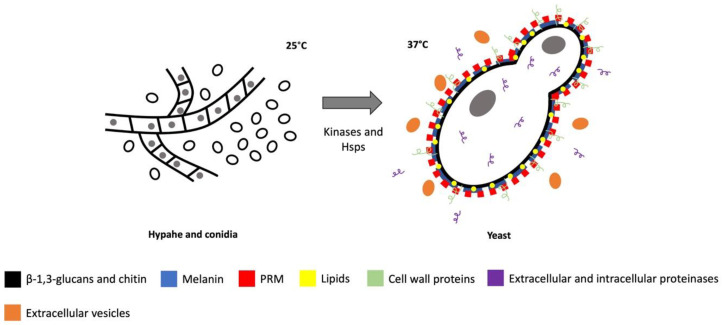
*S. schenckii* virulence factors.

A virulence factor is defined as an element of the pathogen that contributes to damaging the host, whose absence causes reduction of fungal virulence without affecting the organism’s growth or fitness [11]. Based on this definition, only a few virulence factors (Figure 1) of *S. schenckii*, the most studied species of the clinical clade, are reported, such as cell wall proteins, involved in the fungal adherence to the host; kinases and heat shock proteins, which participate in thermotolerance and dimorphism; extracellular and intracellular proteinases, which help the fungus to colonize the host tissues; melanin, which protects the fungus against environmental stresses; extracellular vesicles, which transport elements needed for virulence outside the cell; lipids, found on the cell wall; and biofilm, a structure that participates in the resistance against antifungals; all of which will be described in this review.

The known host immune mechanism against the main *S. schenckii* virulence factors have been thoroughly reviewed elsewhere, for which they will be not discussed herein. Garcia-Carnero et al. (2018) [1] and Ruiz-Baca et al. (2021) [12] offer a broad and in-depth summary of the immune response induced by *S. schenckii.*

## 2. Cell Wall Proteins

The fungal cell wall plays an essential role during the host-pathogen interaction, besides participating in the cell wall integrity and remodeling [13]. Several pathogen-associated molecular patterns (PAMPs) can be found in the cell surface, which might be recognized through pattern recognition receptors (PRRs) of the host immune system, or which function as virulence factors, participating during the infection with a role in the organism pathogenicity [1,14,15].

These proteins are highly glycosylated and can be classified as typical and atypical proteins [16], depending on their secretion mechanisms [17,18,19].

In pathogenic fungi, many glycoproteins participate during the infection, having a role in how the pathogen invades the host, promotes its survival, and evades the host immune response [14,15]. Therefore, protein secretion is important for the pathogenic processes, and can take place by three main mechanisms: (i) co-translational translocation, through the endoplasmic reticulum and Golgi apparatus, which requires the presence of a signal sequence (signal peptide) in the N-terminus of the protein; (ii) post-translational translocation, which is SRP (signal recognition particle)-independent; and (iii) as cargo of extracellular vesicles (EVs) [17,18,19].

Typical cell wall proteins are those with a signal peptide in their N-terminus, which get covalently linked to the cell wall by different bonds to fulfill their biological function [19,20]. Most of these proteins have a glycosyl phosphatidyl inositol-anchor (GPI) that can be either linked to the cell wall or attached to the plasma membrane [19,21,22]. These proteins have a common structure: the peptide signal, domains for ligand binding, an optional central Ser/Thr rich glycosylated region, and a hydrophobic domain at the C-terminal that gets cleaved off and replaced with the GPI-anchor [20,22].

On the other hand, atypical proteins have also been found in the cell wall of many organisms, which neither present the signal peptide needed for the classical secretion pathway nor the GPI-anchor, but instead, they are secreted by non-classical mechanisms, such as post-translational translocation or EVs, and remain non covalently bound to the cell wall [19]. These proteins are known as moonlight, and they can be highly glycosylated and are usually intracellular proteins with enzymatic activities that have different functions on the cell wall, many of which have been found to play important roles in the virulence of pathogenic fungi [23].

The proteins from the cell wall of *Sporothrix* have not been well characterized, but there are several reports about some adhesins that bind to the host extracellular matrix (ECM) proteins, such as fibronectin, laminin, and type II collagen (Table 1) [24,25,26]. Adhesion of the pathogen to the host cells is essential for its correct colonization and further dissemination [27].

Some *S. schenckii* cell wall proteins that bind to fibronectin have been identified, although only a few have been well characterized. These proteins were reported to be of different molecular masses of 152, 92, 70, 67, 55, 50, 44, and 37 kDa, varying among isolates [27].

The most characterized adhesin of *Sporothrix* is the Gp70, found on the three clinically most important species, *S. brasiliensis*, *S. schenckii*, and *S. globosa* [28]. Gp70 is a highly glycosylated typical cell wall protein [29] that can present a molecular weight in a range of 60–70 kDa, probably due to changes in the glycosylation pattern, and with a 3-carboxymuconate cyclase domain that provides the enzymatic activity to degrade aromatic derivates from the environment when *Sporothrix* is growing as a saprophyte on the vegetal matter and debris [29]. This protein has been found attached to the cell wall, but also as secreted and as part of EVs [28,30]. The expression of this protein has been related to the virulence of the species and strains; in highly virulent *S. brasiliensis* isolates, the presence of Gp70 was poorly observed, while in low virulent *S. brasiliensis* and *S. schenckii* isolates, a higher density of the protein in the cell wall was found [30]. The importance of this protein for *Sporothrix* pathogenesis has been widely studied, suggesting that it has a dual role: adhesion and immunogenicity [31]. Due to its lower expression in most pathogenic isolates, and the fact that is the major antigenic protein in the cell wall [30,32,33], it is thought to contribute to the immune response against the fungus, as it was observed by the protective effect of passive immunization with monoclonal antibodies against Gp70 during infection, causing a decreased number in the CFUs [31], and by the fact that sera from all infected patients recognize the protein [34]. On the other hand, its adhesion properties were attributed since the purified protein is capable of binding extracellular matrix proteins, like fibronectin, laminin, and type II collagen [27,31], and treatment of endothelial EMC with antibodies against this protein decreased the attachment of *Sporothrix* yeasts [31]. In addition, it was found that Gp70 contributes to the fungal adherence to mouse tail, since anti-Gp70 antibodies reduced adhesion to the host tissue [34].

Another element of the *S. schenckii* cell wall is the peptidorhamnomannan (PRM), a glycoconjugate composed of mannose (57%), rhamnose (33.5%), proteins (14.2%), and galactose (1%) [35]. Although the PRM has been identified as one of the main antigenic components of the cell wall and, despite its carbohydrate moiety, has been widely studied [35,36,37,38,39], the proteins that compose it were only recently identified [40]. By mass spectrometry, it was found that this complex is composed of 325 proteins, most of which lack signal peptide and, therefore, could be moonlight proteins transported to the cell surface by non-canonical secretory pathways [40,41]. Two of these PRM proteins, Hsp60 and Pap1, were identified as adhesins that bind to different ECM proteins in the host cells [40]. Hsp60 is a chaperone highly conserved in all living organisms and upregulated in response to stress conditions, playing an important role in the cell housekeeping functions [42]. This protein has also been found on the cell wall of other pathogenic fungi, such as *Histoplasma capsulatum* [43], *Paracoccidioides brasiliensis,* and *Paracoccidioides lutzii* [44,45], as immunodominant antigens and participating as virulence factors by giving the fungus adhesion properties [43,45,46].

In *S. schenckii*, it was found that Hsp60 binds to laminin, elastin, fibrinogen, and fibronectin (Table 1). Correspondingly, this protein participates in the fungus virulence, as observed in *Galleria mellonella* assays. When *S. schenckii* yeasts were opsonized with antibodies against the recombinant Hsp60 (rHsp60) and then used to cause infection in the larvae, the fungus lost its ability to kill the host, probably due in part to an increase in the elimination of the pathogen, since the CFUs were significantly reduced. Additionally, this led to an increased humoral immune response, since hemocytes levels, phenoloxidase activity, and melanin production were also increased in this model, which was also probably due to a loss of the adhesion properties of the fungus. In addition, when the rHsp60 was inoculated in the larvae prior to a lethal challenge of *S. schenckii*, in concentrations equal or higher than 40 μg, an increase of the host survival was observed, thus representing a prophylaxis method for the protection against infection by *S. schenckii* [40]. Similar to the Hsp60, the Peptidorhamnomannan-associated protein 1 (Pap1), an uncharacterized protein present in *S. brasiliensis* but not in *S. globosa*, was found to lack a signal peptide and to function as an adhesin. Pap1 binds to laminin, elastin, fibrinogen, fibronectin, and type I and type II collagen and, like Hsp60, participates in the *S. schenckii* virulence. *G. mellonella* assays demonstrated that when yeast cells are opsonized with antibodies against the recombinant Pap1 (rPap1) and that when the rPap1 is used as prophylaxis, the fungus is incapable of killing the host [40].

It is also noteworthy to mention that the genes that code for both proteins were found to be upregulated in the yeast parasitic morphology and when the yeast interacts with HeLa cells, confirming their role during the interaction with the host [40].

## 3. Kinases and Heat Shock Proteins

As in other pathogenic fungi, dimorphism is an important virulence factor for *S. schenckii* (Figure 1) [10], since the fungus needs to change from its saprophytic phase to its parasitic phase, the yeast morphology, in order to grow and disseminate in the host [47]. It has been demonstrated that calcium is highly related to *S. schenckii* dimorphism, stimulating the fungus morphology transition to adapt to environmental changes [48,49]. By binding to calmodulin (CaM), calcium activates Ca^2+^/calmodulin-dependent protein kinases (CaMKs), which are serine/threonine proteins with two major domains, a highly conserved N-terminal catalytic domain and a C-terminal regulatory domain [50,51].

The *S. schenckii sscmk1* gene codes for a CaMK, named SSCMK1, of 407 amino acids with a molecular weight of 45.6 kDa and with the 12 conserved subdomains needed for its function [51,52]. Furthermore, a pak-box/p21-rho-binding domain was found in the protein, which is involved in the regulation of the MAPK pathway that participates in the control of morphogenetic and proliferative processes. In addition, this protein is homologous to members of the CaMK family in other fungi, with a high degree of conservation [51]. Inhibition assays in *S. schenckii* using inhibitors of the CaMK activity, such as W-7, KN-62, and lavendustin C, demonstrated that the yeast is incapable of reentering into the budding cycle. These compounds have different action mechanisms with different degrees of specificity towards the enzyme: W-7 is a calmodulin inhibitor [53]; KN-62 is the most specific inhibitor, affecting the CaM binding domain [54], and lavendustin C inhibits CaMKs as well as tyrosine kinases [55]; yet, with all of the inhibitors used, an increase of the yeast to mycelium transition and an inhibition of the re-entry into the yeast cell cycle was observed, which suggest that the CaM kinase activity and the participation of calcium are needed for the G1/S transition during the budding cycle and are important in the regulation of *S. schenckii* dimorphism [51].

The *sscmk1* gene was later silenced by RNAi and the results obtained by the inhibitors were confirmed with the mutants obtained. These mutants were incapable of growing efficiently in the yeast morphology at 35 °C, growing mainly as mycelium, which is suggested to be due to the decreased expression of the SSCMK1 [49]. In addition, a two-hybrid analysis of proteins that interact with the SSCMK1 identified an Hsp90 homolog that interacts with the kinase [49]. Hsp90 is a protein with essential housekeeping functions for the cell, including the promotion of folding and refolding of proteins during heat shock conditions and proteotoxic stress. This protein has been reported to bind to many client proteins, including kinases/phosphatases related to calcium, in other fungi like *Candida albicans* and *Aspergillus fumigatus* [56]. It was observed that SSCMK1 binds to the C-terminal domain of the *S. schenckii* Hsp90, which might cause an alteration of the Hsp90 activity that induces the release of effector proteins that normally bind to the Hsp90 N-terminal domain, such as calcineurin and other kinases that function as effectors for dimorphism. Therefore, regulation of the Hsp90 by SSCMK1 is suggested, and thus, decreasing the SSCMK1 levels is thought to affect the activity of Hsp90, making the cell more vulnerable to temperature changes [49]. To confirm this, the inhibitor geldanamycin was used to inhibit the Hsp90 activity, which caused an abnormal mycelial morphology, similar to that observed in the sscmk1 silencing mutants. Altogether, these results support the fact that SSCMK1 is necessary for the correct functioning of Hsp90 and, therefore, for *S. schenckii* thermotolerance and dimorphism [49].

Another histidine kinase, DRK1, has also been suggested to participate in *S. schenckii* dimorphism. The cDNA sequence of the DRK1, named SsDRK1, was found in the fungus, with an open reading frame of 4071 bp that codes for a protein of 1356 amino acids of 147.3 kDa [57]. This protein is predicted to be a soluble histidine kinase without transmembrane segments and with three domains: a sensor domain, a linker domain, and a functional domain. This hybrid histidine kinase is an important regulator of dimorphism in other pathogenic fungi such as *Blastomyces dermatitidis* and *H. capsulatum* [58], and since the SsDRK1 has a 65% identity match to that of *B. dermatitidis* and is overexpressed in the yeast morphology, it was suggested to be involved in the *S. schenckii* dimorphism [57]. This was later confirmed by the silencing of SsDRK1. The silencing mutants presented retarded growth in both mycelial and yeast morphologies, demonstrating a delay in asexual development. Likewise, yeast cells had abnormalities in their ultrastructure, with the presence of short and abundant plasma membrane invaginations, while conidia and hyphae had irregular morphologies, with increased and variable cell wall thickness, lack of the electron-dense zone on the cell wall surface, and plasma membrane invaginations [59]. In addition, the mutants had an increased sensitivity to congo red and zymolyase, and defects in colony pigmentation were observed since the mutants failed to produce as much melanin as the wild type strain. All of these results suggest that a decrease in SsDRK1 levels is associated with decreased polysaccharide levels in the *S. schenckii* cell wall [59].

Further, the silencing mutants had decreased virulence in a murine model of cutaneous sporotrichosis, since mice infected with these strains had reduced inflammatory cell infiltration, such as neutrophils and mononuclear cells, in the lesions, which might be explained by the deficiencies in asexual development and dimorphism, cell wall composition and integrity, and melanin synthesis [59].

## 4. Extracellular and Intracellular Proteinases

For pathogenic fungi, proteinase production for enzymatic digestion of the host tissues is essential for cell growth and infection development, and several of these enzymes have been suggested in *S. schenckii*. It was observed that when the yeast morphology of the fungus grows in media with albumin or collagen as nitrogen sources, it produces extracellular proteinases. Two of these were isolated and characterized, Proteinase I and Proteinase II. Proteinase I is a serine proteinase, with a molecular weight of 36.5 kDa, and an optimal working activity at pH of 6.0, with chymotrypsin like characteristics, while Proteinase II is an aspartic proteinase, with a molecular weight of 39 kDa, and an optimal working activity at pH of 3.5, with cathepsin D-like characteristics. Hemoglobin was the best substrate for both enzymes, but these were also capable of hydrolyzing stratum corneum, type I collagen, and elastin [60,61]. In vivo expression of these proteins was proved with a murine model of intracutaneous infection, where high titers of antibodies against both proteinases were found during the whole infection period [61], and treatment of the lesions with the proteinases’ inhibitors, chymostatin and pepstatin, reduced nodule development and caused faster regression [62]. These results suggest that Proteinase I and II help *S. schenckii* to degrade skin constituents, multiply in the dermis, and invade the host [60,61,62].

In addition, the secretion products of an *S. schenckii* pure yeast preparation were analyzed, demonstrating the presence of highly immunogenic proteins with molecular weights between 40 and 70 kDa, some of them with proteolytic activity. Although Proteinase I and II are thought to be present in this extract, proteases that were capable of cleaving different subclasses of human IgG were also found, which confirms the presence of different proteases activities performed by *S. schenckii* [63].

On the other hand, the presence of intracellular proteolytic activity in the yeast morphology was also reported. At pH of 5.0, proteolytic activity was observed and associated with proteins between 200 and 116 kDa, while at pH 7.0, this activity was even higher and associated with proteins between 200 and 70 kDa. It was demonstrated that these proteinases induce actin cytoskeleton alteration in epithelial cells, since cells treated with these enzymes showed a decrease of cytoplasmic actin stress fibers, producing spherical-shaped epithelial cells, membrane blebbing, and cytoplasmic actin filament alteration. This proteolytic activity was then inhibited with cysteine or serine protease inhibitors, which blocked the disruption of the actin stress fibers, confirming the proteinases substrates. These results suggest the presence of intracellular proteinases that alter the morphology and structure of the host cells, which also promotes the host tissue colonization by *S. schenckii* [64].

## 5. Melanins

Melanins are a class of pigments generated by oxidative polymerization of phenolic compounds, and although those pigments are not essential for the growth of the fungus, they play a fundamental role in the organism survival, conferring stability against certain environmental stressors, such as UV radiation and host defenses [65,66]. Although different biosynthetic routes are used for melanin production, in the case of fungi, the DOPA and DHN routes are the ones that predominate, and it has been shown that *S. schenckii* uses both routes. The first pathway requires the presence of exogenous substrates, such as 3,4 dihydroxyphenylalanin, and the melanin produced is known as eumelanin, while the second route can use precursors produced via acetyl-CoA from the glycolysis. Furthermore, there is a third pathway employed by *S. schenckii* called pyomelanin that uses L-tyrosine precursors for the melanin synthesis employed by *S. schenckii* [67,68,69,70]. Melanins confer great advantages on the organisms that possess them, and a feature that we will highlight is their role as a virulence factor in the pathogenic fungus *S. schenckii*. For instance, nonmelanized cells of *S. schenckii* are more susceptible to being engulfed by human monocytes and murine macrophages [67]. Melanin produced by the DHN route is of greater quantity and faster production in *S. brasiliensis* compared to *S. schenckii*, which correlates with the greater virulence that *S. brasiliensis* presents in the murine model, demonstrating the impact that the pigment has on the virulence of *Sporothrix* [71]. Furthermore, melanin protects fungal cells against reactive oxygen species (ROS) and nitric oxide (NO), chemical compounds released by host cells as a defense mechanism. Moreover, this pigment has been proposed as a scavenger that protect the fungus against UV radiation [67]. The mutant strain of *S. schenckii* lacking melanin causes less progression of sporotrichosis in the murine model compared to the wild type strain; therefore, it has been proposed that the presence of melanin in *S. schenckii* may be an important factor in the development of the mycosis [72]. In the human pathogenic fungi *H. capsulatum*, *Cryptococcus neoformans,* and *P. brasiliensis*, the melanin acts as a fungal component that provides resistance to the antifungal drugs amphotericin B and caspofungin [73]. The same role has been demonstrated for *S. brasiliensis* [70]. In addition, melanized cells of *S. schenckii* and *S. brasiliensis* have shown resistance to the antifungal drug terbinafine, another of the drugs of choice to treat sporotrichosis [69]. As an antifungal resistance factor, melanin and its synthesis pathways could be a target for the design of new antifungal drugs for sporotrichosis therapies. A recent study has shown that melanin is a key factor to prevent phagocytosis of *S. globosa* by THP-1 macrophages, given that this cell wall component neutralizes the respiratory burst and suppresses the ROS and NO production; besides, the presence of melanin in *S. globosa* decreases the expression of TLR2 and TLR4, receptors involved in the recognition of the fungus [74,75,76]. Therefore, the presence of a said component in the cell wall of the pathogen reduces the possibility of the host to fight the fungus. Finally, the pro-inflammatory response is also decreased, especially TNFα and IL-6, in the presence of *S. schenckii* wild-type fungus compared to the mutant lacking melanin [74].

## 6. Extracellular Vesicles

Extracellular vesicles, lipid bilayer structures released by cells, have been described in a variety of human pathogenic fungi such as *C. albicans*, *Candida parapsilosis* and *H. capsulatum;* in the last one, the vesicular content has molecules involved in the cell wall architecture, but also includes phospholipids and proteins related with virulence, pathogenesis, and stress response [77]. The formation of said structures has been described in *S. schenckii* [77], and presumably, the macromolecules in this vesicular transport contain products that are associated with the pathogenesis of the fungus. In a recent proteomic analysis of *S. schenckii* EVs, 40 different proteins were found, of which 35% were not characterized; in the case of *S. brasiliensis*, the EVs harbor a variety of 63 proteins, 27% of which are not characterized. However, in both species, most of the characterized proteins are related to metabolic process, transport, oxidation-reduction reactions, stress response, DNA metabolic process, and cellular component organization [78]. The EVs of *S. brasiliensis* activate the production of the cytokines TNFα, as well as INFγ and the interleukins IL-12p40, and IL-1β, in BALB/c mice [78]. This suggests that EVs participate as virulence factors in the establishment of infection.

## 7. Lipids and Biofilm

Lipids associated with the cell wall of *S. schenckii* are involved in pathogenesis since these components inhibit the phagocytic process by macrophages of Swiss mice. However, lipids can also trigger a high release of NO and TNFα, which help to eliminate the pathogen and to stimulate the immune response [79].

Biofilm formation has been considered a virulence factor in different human pathogenic fungi such as *Candida*, *Aspergillus* and *Cryptococcus* [80,81,82], and recent studies have shown that *S. schenckii* is capable of generating this complex structure, whose main components are glycoproteins, polysaccharides, nucleic acids, and lipids [83,84,85]. This structure complicates antifungal treatment since, apart from being a barrier that prevents the access of certain molecules to the interior of the biofilm, it is a key participant in the acquisition of resistance to the antifungals of choice, such as itraconazole, fluconazole, voriconazole, posaconazole, amphotericin B, flucytosine, and caspofungin [84,85,86,87]. The little access that antifungals have to reach their target cells, either by impediment, adsorption, inactivation, neutralization, or expulsion caused by the biofilm, causes the mycosis to become recalcitrant, for which biofilm development has become an important factor for the development and persistence of sporotrichosis.

**Table 1:** Known *S. schenckii* virulence factors.

*S. schenckii* Virulence Factors		Function	References
Cell wall proteins	Gp70	Adhesin that binds to fibronectin, laminin, and type II collagen Immunodominant antigen	[27,30,31,33,88]
	Hsp60	Adhesin that binds to laminin, elastin, fibrinogen, and fibronectin	[40]
	Pap1	Adhesin that binds to laminin, elastin, fibrinogen, fibronectin, and type I and II collagen	[40]
Kinases and heat shock proteins in dimorphism and thermotolerance	SSCMK1	Morphological switching and thermotolerance	[49,51]
	Hsp90	Response to heat shock and proteotoxic stress Thermotolerance	[49]
	DRK1	Morphological switching and thermotolerance	[57,59]
Extracellular and intracellular proteinases		Degradation of skin constituents and cleaving of antibodies	[60,61,62]
Melanin		Protection against environmental stresses and phagocytosis Neutralization of reactive oxygen species and nitric oxide Resistance to antifungals	[67,69,70,72,73]
Extracellular vesicles		Transportation of molecules involved in pathogenesis	[77,78]
Lipids		Protection against the immune response and phagocytosis	[30]
Biofilm		Resistance to antifungals	[79,85,87,89]

## 8. Conclusions

Some human pathogenic fungi possess virulence factors that give them the ability to cause disease in the host. Despite the immune response that is triggered to combat these pathogens, they survive and persist in the host thanks to virulence factors that help them fight and damage host tissue. The study of *S. schenckii* virulence factors is important to continue building knowledge about sporotrichosis, and to continue with the development of new drugs to combat mycosis, as well as the possibility of generating vaccines that allow the prevention of the infection.

## Data Availability

Not applicable.
